# The Transformative Power of Embodied Behaviour: Influencing Tourists’ Experience in the Guangzhou Marathon as a Mass Participant Sports Event

**DOI:** 10.3390/bs15010090

**Published:** 2025-01-20

**Authors:** Xin Xu, Guangquan Dai

**Affiliations:** Department of Tourism Management, South China University of Technology, Guangzhou 510006, China

**Keywords:** mass participant sports event, event tourism, embodied experience, event attachment, Guangzhou Marathon

## Abstract

Mass-participation sports events (MPSEs) are of significant value to the fields of sports, culture, and tourism. MPSEs have witnessed a remarkable surge in popularity, which has led to a complex interplay of factors influencing participants’ overall experience, making it crucial to understand the role of embodied behaviour. However, the existing literature is deficient in terms of providing substantial evidence, particularly with regard to the growing significance of experience planning as a core aspect of event design. This research employed the Guangzhou Marathon, one of the most renowned MPSEs in China, as a case study. The objective of this research is to investigate and extend the knowledge of the embodied behaviour of MPSE tourists through the mixed method of a questionnaire survey together with interviews. Furthermore, this research aimed to explore the antecedents and consequences of the embodied experience formation process. The findings demonstrated the pivotal role of the embodied experience in shaping tourist perceptions and subsequent intentions. Specifically, this research suggested that tourist behaviour with experience and attachment influenced the perceived value and cost of participation willingness through moderating and mediating effects. The findings contribute to the existing knowledge on sports tourism and behavioural studies and provide sustainable event management strategies.

## 1. Introduction

Sports event tourism has gained significant attention due to the increasing number of people travelling to different destinations to attend sports events (Getz & Page, 2019). It offers tourists a unique and exciting experience, allowing sports fans to witness their favourite games and athletes and even participate in games themselves. From the perspective of destination development, sports event tourism generates an economic boost for the host city/country through ticket sales, accommodation, transportation, and other related tourist expenses (Sato et al., 2015; Wegner et al., 2015). Furthermore, it helps to promote the destination image by showcasing its culture, attractions, and society to a diverse audience. This has led to travelling to participate in sports events becoming a new trend and phenomenon (Ramchandani et al., 2017).

Among all sports event types, compared to spectator sports events such as the Olympic Games, mass participant sports events (MPSEs) are particularly noteworthy. MPSEs are sports events that encourage broad public engagement, with the characteristic of thousands of participants for a single event. However, MPSEs are of significance not only by their scale but also by addressing the potential to generate a healthy lifestyle. MPSEs provide sports fans with the opportunity to be fully immersed in the whole event process. The typical MPSEs are marathons, half marathons, triathlons, cycling races, etc. However, as only a few sports can be organised on a mass scale, only certain participants can enjoy the games both as event participants and tourists. Their experience, especially their tourist experience, has long been ignored.

This research aims to examine the complex nature of the individual tourist’s embodied experience, including its antecedents and consequences, within the context of culture- and society-embedded MPSEs. Despite the growing popularity of experience-based tourism, MPSEs face challenges due to commercialisation and modernisation by event organisers, resulting in a lack of uniqueness in experience planning and design (Du et al., 2015). MPSEs have gained popularity since the late 20th century in China. During the development of MPSEs, both the government and the market have tried to profit from the event while underestimating the initiative and contribution of the participants. Participants are of the utmost importance to the events, and they, in turn, promote a better event (Getz & Page, 2019). In the Chinese context, the organisation of MPSEs involves diverse purposive stakeholders, among whom tourists are often paid less attention (Cohen & Cohen, 2019).

The existing literature on the tourist experience in sports events has mainly focused on individual behaviour based on personal background and intention (Risitano et al., 2017). While an event itself is a tourist attraction as well as a tourist destination, the whole event experience, quite similar to the tourist experience, is a dynamic process. Based on the event phases, the event experience can be divided into the pre-event experience, during-event experience, and post-event experience (Getz & Page, 2019; Lin & Kuo, 2016). The seasonality, liminality, and cultural content of event features can strongly influence the event experience (Lin & Kuo, 2016; Risitano et al., 2017). The experience in MPSEs is often assessed by main determinants such as perceived value which reflect the cognition of the events (Lin & Kuo, 2016; Neuhofer et al., 2020). However, although the experience has become more diverse and personalised, the internal mechanism of how tourist attitudes and behaviours are influenced by the emotional and embodied experience in MPSEs is still undefined.

The concept of embodied experience originated from embodied cognition theory (ECT). ECT is promoted by Varela, Thompson, and Rosch (Varela et al., 2017), and it suggests that cognition is shaped by the state and capacity of the body, including memory and the body’s interaction with the environment. The embodied experience always refers to the understanding and perception of the world through the physical body and sensory experiences (Varela et al., 2017). In the tourism context, ECT is applied to study the interaction among the body, cognition, and situation, such as tourist destinations and events (Jiang & Yu, 2020). The tourist’s embodied experience is frequently deemed to describe how tourists engage with their surroundings through their emotions and bodies, leading to a more personal connection (Jiang & Yu, 2020). In general, the more the body and emotions are involved in tourist behaviour, the deeper the experience could be (Cutler & Carmichael, 2010; Knobloch et al., 2017). The embodied experience acts as an important predictor of tourists’ attitude and participation in tourism (Cutler & Carmichael, 2010; Getz & Page, 2019; Knobloch et al., 2017; Lin & Kuo, 2016). Interestingly, even when the embodied experience is quite painful or negative, for example, in the context of dark tourism, it can have positive outcomes and can contribute to building the meaning of life (Nawijn & Biran, 2019).

For the last few decades, event studies have focused on the participation experience (Getz & Page, 2019). While the previous literature addresses the general factors influencing the overall experience, the research fails to consider the role of the body and emotion played in the dynamic process of experience construction (Xiang & Cheah, 2023). By examining the body involvement, emotional function, significance of the situation, and their intricate interaction in the process of constructing experience in the context of events, this research broadens the existing knowledge of the literature and provides valuable insights into the multi-dimensional dynamics underlying tourist behaviour towards attending MPSEs.

**Figure 1:**
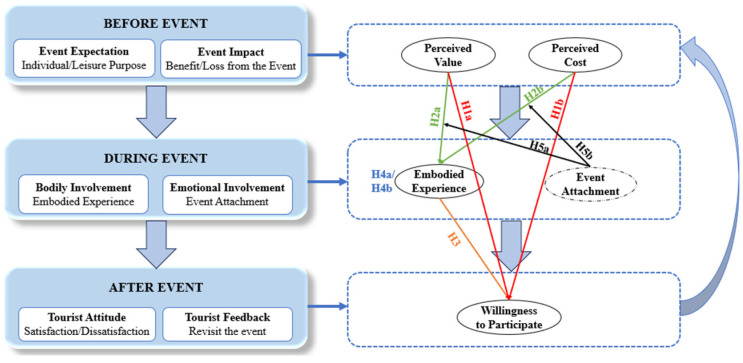
Theoretical framework.

This research employed qualitative methodology to investigate the construction of the event experience through the event phases, especially the influence of the embodied experience and event attachment. The research drew upon the theoretical framework of embodied cognition theory (ECT) to develop a research framework, as illustrated in Figure 1. The framework demonstrates the factors that happen in each phase of the building of the tourist experience in MPSEs, including the perceived value, perceived cost, willingness to participate, embodied experience, and event attachment. The findings will provide both theoretical and managerial implications for sustainable event management in the future organisation of MPSEs and management for event host cities, as well as event industries.

This research focused on a typical MPSE held in the southern part of China, the Guangzhou Marathon. It is the fastest-developed marathon in China and is now the World Athletics Gold Label Road Race since 2018. This research provides a comprehensive case study of the Guangzhou Marathon to further explore the organisation of MPSE in the Chinese context, as it is usually complex.

This research aimed to contribute to the theory in two main areas. Firstly, the significance of the body, as well as emotion, in the sports event experience is addressed in this research, exceeding the economic and social functions. By emphasising the body and emotion dimensions in tourists’ construction of the event embodied experience, this research strengthened the theoretical framing of body involvement and emotional factors in the scope of MPSE research. Consequently, it also offers a comprehensive aspect to understand the tourism value of MPSE from the embodied cognition theory. Secondly, a model of the experience process was developed to illustrate the tourists’ embodied experience within mass participant sports events. Through field observation of MPSE and the theoretical model test, the mediating role of the body and the moderating role of emotion were unveiled. The research has demonstrated how the embodied experience, as the key factor in the event process, affects or is affected by other core concepts in the event context, for example, perceived value, perceived cost, willingness to participate, and event attachment. The findings provide knowledge for the advancement of MPSE research, expanding the internal and external factors shaping tourists’ event participation.

## 2. Literature Review

### 2.1. The Influence of Tourist Event Perception on Their Behaviour

Research on event perception has received considerable attention (Getz & Page, 2019; Nawijn & Biran, 2019). The tourist perception of a tourist destination is confirmed based on the culture and environment. Similarly, the perception of an event is often impacted by the event design and situation, which is of great value (Getz & Page, 2019; J. Zhang & Dai, 2023). Prior experience of similar events will influence the tourist’s decision to participate in the current event, as well as their subsequent evaluation of it (Andrades & Dimanche, 2018). The term ’event perception’ is typically used to describe the tourist’s overall assessment of the value and cost of the experience they have gained from the event (Andrades & Dimanche, 2018). The perceived value of an event represents the tourist’s overall assessment of the experience they have gained from it, whereas the perceived cost refers to the amount paid or given to obtain this experience. Both perceived value and cost relate to the expense of the obtained experience.

Meanwhile, studies have demonstrated that tourism events have resulted in a shift in focus towards the body among tourists (Getz & Page, 2019). In comparison to conventional tourist behaviour based on sightseeing, tourist behaviour has diversified due to the advent of more immersive on-site experiences. Some researchers have put forth the proposition that, given that tourism is an interactive behaviour between the body and the environment, embodiment should be employed in the case of analysing experiences that are highly body-oriented (Lu et al., 2020; Xiang & Cheah, 2023).

The previous literature has found a relationship between tourists’ perception and their behaviour, including their willingness to participate in the tourism process and their embodied experience. For example, when the perceived value obtained is positive for a certain destination, tourists are more willing to visit and create a positive experience (Nawijn & Biran, 2019). Likewise, in an event, the more positive the perception that the tourists have, the more immersive the embodied experience will be in the event (Nawijn & Biran, 2019). When tourists think a festival will provide benefits and create more value, they are more likely to participate, and if they feel the festival design is bad, they usually refuse to attend (J. Zhang & Dai, 2023). The perceived value of an event can be enhanced by increasing the perceived benefit while decreasing the perceived sacrifice. The perceived cost of an event can be evaluated by examining the actual monetary and non-monetary expenses (Ahn & Kwon, 2020). Therefore, based on the existing literature, this research reached the following hypotheses:

**Hypothesis** **1a.** *Tourists’ perceived value has a positive effect on their willingness to participate*.

**Hypothesis** **1b.** *Tourists’ perceived cost has a negative effect on their willingness to participate*.

Embodied cognition theory (ECT) has been well noted for its application to examining the embodied experience in tourism and events. The main concepts in the ECT theoretical framework are the body, cognition, and situation, and the embodied experience is characterised by bodily involvement, including thinking, emotions, perceptions, and sensory experience. The literature has provided evidence that the tourist’s embodied experience is shaped by their comprehensive understanding and assessment of the tourism process as well as its impact. Empirical studies have demonstrated that tourists’ perception has a significant impact on their experience at tourist destinations, and the on-site experience in turn could influence future decision-making (Lv et al., 2022; H. Zhang et al., 2016). The majority of research exploring the relationship between tourist perception and their experience has confirmed that tourist perception does influence experience. Yet, when it comes to the embodied experience including features such as cognitive responses, sensory factors, and effectiveness, the relationship is undefined. Based on embodied cognition theory, this research makes the following hypotheses:

**Hypothesis** **2a.** *Tourists’ perceived value has a positive effect on their embodied experience*.

**Hypothesis** **2b.** *Tourists’ perceived cost has a negative effect on their embodied experience*.

### 2.2. The Influence of Tourist Behaviour on Their Attitude

Event processing plays a key role in shaping the tourist’s impression, both during and after their experience at the destination. The relationship between tourist behaviour and attitude is complex and dynamic. The actions and experiences of tourists can influence their overall perspective and feelings towards a destination or experience (Tussyadiah, 2014). A tourist who has a deeply engaging experience with a destination is more likely to take part in future events. Research has shown that tourists who are actively involved in local activities and have more experience are more likely to be more willing to share information with others (Andrades & Dimanche, 2018). In the event context, the event experience can be achieved by the event organiser providing unique services or unique scenarios to event participants. While the relationship between tourist behaviour and their attitude has been explored, the majority of research confirmed the link within the event experience process. When more experience is generated and formed at the event, their willingness to participate in the event tends to be higher (Baker et al., 2018; Du et al., 2015; Jiang & Yu, 2020).

The willingness to participate in tourism activities has emerged as a crucial factor in shaping the tourist experience (Mandagi et al., 2024; J. Zhang & Dai, 2023). Previous research has demonstrated that when tourists actively engage in events rather than remain passive observers, their experience levels tend to increase significantly (Tsuji et al., 2007). The willingness to participate and the tourists’ real participation create a sense of involvement and personal investment in the experience, leading to higher levels of enjoyment and fulfilment. Tourist willingness to participate represents their readiness to engage in activities, interact with local culture, and immerse themselves in the event experience (Chen & Rahman, 2018). Studies have shown that tourists who demonstrate greater satisfaction levels with their overall experience tend to report higher willingness to participate (Chen & Rahman, 2018; Prebensen & Xie, 2017; Tsuji et al., 2007).

While the embodied experience has been discussed in tourism and event research in recent decades, the importance of this overall experience is well-recognised in shaping tourists’ attitudes (Y. Ouyang et al., 2021; Xiang & Cheah, 2023). The embodied experience involves multi-sensory engagement, including visual, auditory, tactile, and olfactory elements (Varela et al., 2017). This sensory experience creates more profound and meaningful physical connections between tourists and their environment. When tourists physically engage with their surroundings, they develop a more intimate understanding and appreciation of the destination or event, enhancing their emotional connection (Y. Ouyang et al., 2021; Xiang & Cheah, 2023). Studies have demonstrated that such embodied experiences contribute significantly to tourist memory formation and their impressions (Farkic, 2022; Y. Ouyang et al., 2021).

The embodied experience is confirmed to influence future attitudes and feedback, and tourists who reported higher levels of embodied engagement were significantly more likely to express intentions to return to the destination. Similarly, it is applied to the event context and event situation through multiple mechanisms (Lu et al., 2020; Wen et al., 2022). For instance, traditional festivals may offer the opportunity to experience and appreciate cultural traditions, and when the tourists are fully immersed themselves in the event, they have more lasting impressions (Xiang & Cheah, 2023). Based on the existing literature, the hypothesis was made as follows:

**Hypothesis** **3.** *Tourists’ embodied experience has a positive effect on their willingness to participate*.

### 2.3. The Importance of Embodied Experience and Event Attachment in MPSE

The concept of embodied experience is regarded as a pivotal element within the domain of tourism and event research. Furthermore, the pivotal role of this multi-sensory experience in shaping tourism outcomes has been identified as a critical element in previous research (Simonsen et al., 2017; Xiang & Cheah, 2023). The willingness of tourists to participate in tourism activity has been identified as a fundamental factor influencing their experience (Lu et al., 2020). Research findings indicate that tourists who demonstrate a higher willingness to engage in physical and sensory experiences are more likely to enhance their participation levels. This supports the mediation effect of the tourist experience (Matteucci, 2016). As tourist experiences become increasingly sensory, they tend to be more multi-sensory and more engaged with the environment (Huang et al., 2024). Similarly, the experience shapes event outcomes in the immersive atmosphere provided by the event situation (Simonsen et al., 2017). For example, research has confirmed that the embodied experience helps the tourist to gain self-identity within the festival (Xiang & Cheah, 2023). Through the perspective of embodied cognition, it is useful to view the relationship of experience in the event and the post-event intention in a dynamic view (Gao et al., 2024). The definition of attachment has evolved significantly from its psychological origins to its applications in tourism and event studies. The attachment itself explains the emotional bonds between individuals and significant others (Z. Ouyang et al., 2017). The definition highlights that attachment styles affect behavioural and emotional responses (Tsaur et al., 2019). The environmental science researchers extended the definition to measure the relationship between the human and the environment and put out place attachment to address the bonds between people and places (Kusumah, 2023). In this way, event attachment is adapted to the relationship of tourists and event context (J. Zhang & Dai, 2023), especially the participants and the event atmosphere. In the role of event attachment, robust attachment can give rise to a profound connection with the event, which may subsequently give rise to a rich array of emotional memories and associations pertaining to the event (J. S. Lee & Kang, 2015; Z. Ouyang et al., 2017; Tsaur et al., 2019).

Event attachment is often considered to have some key factors which are formed by the event participants while they are attending the event, for example, self-identity, place dependence, etc. (J. Lee, 2014; Xiang & Cheah, 2023). As event attachment is confirmed to affect whether tourists participate in the event or not, it will subsequently affect their behaviour and emotions during the event (Kusumah, 2023; J. Lee, 2014; Savinovic et al., 2012). Following the process of emotional involvement, event participants internalise the event and event environment into their self-concept by undergoing a series of progressive steps, beginning with awareness, progressing to attraction, and culminating in allegiance (Getz & Page, 2019; Tsaur et al., 2019). Some researchers have explored the role of event attachment when it acts as the emotional function in tourism and events (J. S. Lee & Kang, 2015; J. Zhang & Dai, 2023). The different strength of the attachment can vary as the intensity of tourist perception and behaviour varies (Mainolfi & Marino, 2020; Y. Ouyang et al., 2021). Event attachment can function as the moderator via the tourists’ process. Participating in a mass participant sports event, for instance, can facilitate the development of a stronger sense of belonging and identity with the event community. This growing social identity forms the basis of event attachment. As tourists engage more frequently, they establish relationships, share experiences, and create memories, all of which contribute to a heightened emotional bond (J. Lee, 2014; J. S. Lee & Kang, 2015). The prevailing Attachment Theory illustrates that individuals with a stronger attachment to an object or event tend to be more forgiving and resilient in the face of challenges (H. Zhang et al., 2016). Thus, in the context of MPSEs, when confronted with heightened perceived costs, such as escalating accommodation prices or diminished complementary provisions, the individuals’ emotional attachment to the event serves as a mitigating factor, thereby diminishing the adverse impact on their subsequent behaviour (J. Lee, 2014; H. Zhang et al., 2016). When faced with a cost increase, like a more expensive registration fee, tourists’ strong event attachment motivates them to look beyond the monetary aspect. They might recall the previous memorable experiences, the support from spectators, and the personal achievements they accomplished on the course. These positive memories and emotional connections override the negative perception of the cost, thereby maintaining or even enhancing their embodied experience (Hallmann & Zehrer, 2024). While it is a significant factor in the construction of tourist behaviour, the influence path from embodied experience and event attachment remains undefined (Kennedy et al., 2019; Xiang & Cheah, 2023). Based on the above literature, this research has promoted the following hypotheses:

**Hypothesis** **4a.** *The embodied experience mediates the relationship between perceived value and willingness to participate*.

**Hypothesis** **4b.** *The embodied experience mediates the relationship between perceived cost and willingness to participate*.

**Hypothesis** **5a.** *Event attachment positively moderates the relationship between perceived value and the embodied experience. For tourists with a higher level of event attachment, the positive impact of perceived value on the embodied experience is stronger, and vice versa*.

**Hypothesis** **5b.** *Event attachment negatively moderates the relationship between perceived cost and embodied experience. For tourists with a higher level of event attachment, the negative impact of perceived cost on the embodied experience is weaker, and vice versa*.

As shown in Figure 1, the conceptual framework was constructed based on the above discussions. To further explain Figure 1, the left side represents the whole process that tourists experience in the event. Before the event starts, tourists have expectations based on their previous resource collection and event experience. They might feel both benefits and losses from the event as they evaluate the cost and value of the event. During the event, bodily and emotional factors will emerge to affect their behaviour. After the event, tourists will form an attitude towards the event, they will feel satisfied or not, and decide whether they will be willing to participate in the event or not. On the right side of Figure 1, different phases of the event process have certain factors to form. The model demonstrates the relationship and mechanism among tourists’ perception, behaviours, and attitude, including perceived value, perceived cost, willingness to participate, embodied experience, and event attachment, and these factors represent the measurable variables for phases corresponding with the left side.

## 3. Methodology

### 3.1. Research Background and Research Design

As one of the most renowned and high-profile marathons in mainland China, the Guangzhou Marathon has been held annually in December since its establishment in 2012. The Guangzhou Marathon has been designated a World Athletics Gold Label Road Race since 2018. The theme of the Guangzhou Marathon, ’Renowned City, Harmony, and Health,’ has integrated national fitness and competitive sports. It is the fastest-developed marathon in China, and has become an emblem of the city’s appearance, social, and cultural identity. In 2024, the Guangzhou Marathon has been held for 11 sessions, with a total of over 300,000 participants. During each session, the event generates a significant amount of revenue related to marathon events, exhibitions, and city tourism. The success of the Guangzhou Marathon has led to the event’s expansion in terms of both influence and reputation. As a mass participant sports event, the Guangzhou Marathon aims to attract not only local citizens to participate, but also tourists and runners from home and abroad to promote the event and sports spirit. Runners have the opportunity to fully immerse themselves in the event atmosphere and make use of everything to generate their experience. In this way, all these features make the Guangzhou Marathon a typical case for the research of MPSE and serve as a platform for the deep involvement of embodiment.

The Guangzhou Marathon 2023 marked the resumption of the event following the postponement caused by the global pandemic. The research was conducted through a large-scale survey, and the questionnaire was designed through a comprehensive literature review and tailored to the specific context of MPSE. A meticulous double-translation process was employed to translate the initial questionnaire into Mandarin Chinese. This procedure involved the input of experts who provided feedback to enhance the language, semantics, and content. One of the researchers is an experienced marathon runner and has access to runners from different cities in China. Through the network built by researchers, the research started with small-scale interviews with different types of runners. The main aim of these front-stage interviews was to find out what is most important to runners when travelling to participate in a marathon event, as well as to gain insights into their previous experiences. After the interviews, corresponding with the existing literature review, the original questionnaire was formed.

A small-scale pilot study was conducted in October–November 2023, when the quota for Guangzhou Marathon 2023 was released. One of the researchers started from the surroundings to conduct the survey and sent the questionnaires to those who were selected to participate in the event. In total, 103 experienced runners completed the survey. The original questionnaire consisted of 20 questions to assess the variables from previous tourism research. Subsequently, an exploratory factor analysis was conducted, and 3 questions were removed because they were not significant for the validity and reliability test. The final questionnaire comprised 17 questions for variables and another 7 questions for demographic basis. The Cronbach’s alpha coefficient of the data was 0.938, indicating high reliability. The results of KMO and Bartlett’s chi-square test, discriminant test, and *t*-test were employed to indicate the validity of the questionnaire. Runners in the pilot study have provided feedback for the questionnaire design and language use. The revised version was used for the formal survey with a more comprehensive understanding and clearer expression.

The formal questionnaire comprised two sections with 24 questions. The initial section employed a seven-point Likert scale to assess the latent variables, ranging from 1 (strongly disagree) to 7 (strongly agree). This section encompassed the dimensions of variables which consist of the whole process including before-event variables: perceived value and perceived cost; during-event variables: embodied experience and event attachment; and post-event variables: future willingness to participate. The embodied experience was measured by 6 items (Lin & Kuo, 2016; Xiang & Cheah, 2023), the perceived value and perceived cost together were measured by 4 items (Nawijn & Biran, 2019; UNWTO, 2008; J. Zhang & Dai, 2023), event attachment was measured by 3 items (Z. Ouyang et al., 2017; J. Zhang & Dai, 2023), and willingness to participate was measured by 4 items (Prayag et al., 2013; Weaver, 2013; Wicker & Hallmann, 2013). These established scales have been shown to be reliable and valid in previous studies conducted by both domestic and international scholars separately. The second section focused on the demographic characteristics and general travel preferences of marathon runners, mainly including gender, age, educational level, income, occupation, the number of participants in the marathon game, the number of years spent running marathons, and other relevant information.

### 3.2. Data Collection and Data Analysis

This research originally investigated the tourists’ embodied experience in the Guangzhou Marathon event. Based on the results of the pilot study results, a large-scale questionnaire survey was applied and conducted for Guangzhou Marathon runners. The questionnaire began with a question of whether these runners were tourists to meet the UNWTO definition of tourists (UNWTO, 2008). They had to stay in the host city for the event and meet the length of stay. The formal survey was conducted from December 2023 to December 2024, during and after Guangzhou Marathon 2023 and 2024. Convenience sampling and snowball sampling were applied to marathon runners. In December 2023 and December 2024, the on-site survey was conducted during the event, at the race finishing line and various parts along the running course on the event day. In total, 500 questionnaires were distributed and 458 were returned, and 412 of them were suitable for data analysis. In January 2024, the online survey was distributed in a runners’ group chat, and those who had confirmed their participation in the Guangzhou Marathon filled out the questionnaire. A total of 208 runners completed the questionnaire and 160 of them were suited for data analysis. After reviewing the validation of data, the questionnaires with missing answers, identical answers for all questions, and a short completion time (online survey only) were excluded. A total of 572 questionnaires were used for the next step of data analysis. The effective response rate was 80.8%.

Within the valid sample, 325 are male, accounting for 56.8%, and 247 are female, accounting for 43.2%. All respondents had completed a full marathon in Guangzhou Marathon and identified themselves to be both runners and tourists. According to the Chinese Athletics Association (CAA) (CAA, 2023), runners are generally divided into 9 age groups. The age distribution is concentrated in the 25–34 age group, with 44.5% of all respondents, followed by 14.3% in the under 24 age group and 10.8% in the 40–44 age group. In terms of education, the majority of respondents have a high level of education, with 49.8% holding a university or college degree and 37.6% holding a postgraduate qualification. The majority of respondents have an income under 6000 RMB per month, accounting for 56.1%, and 23.4% have an income above 10,000 RMB per month. In terms of occupational distribution, all general categories in the Classification Catalogue of Various Occupations in the People’s Republic of China (PRC) are represented in the questionnaire (National Occupational Classification Code Revision Committee, 2022). While 64.2% have attended marathons for 2–6 years, 31.5% have participated in marathons 10 or more times, followed by 26.9% who have participated in marathons 7–9 times. The sample data were found to be reliable in terms of gender, age, education level, and income, which is consistent with runners’ data released by the CAA (CAA, 2023).

## 4. Findings

### 4.1. Validity, Reliability, and Confirmatory Factor Analysis

SPSS 26.0 was employed in the questionnaire data to exhibit the reliability. The result showed that the Cronbach’s alpha coefficients of different variables ranged from 0.817 to 0.920, and the total Cronbach’s alpha coefficient was 0.956, which suggested a satisfactory reliability for data analysis. The KMO and Bartlett’s chi-square test showed that the KMO value was 0.903, and the Bartlett’s test of sphericity was found to be significant, suggesting the effectiveness of the exploratory factor analysis. The principal component analysis pointed out that the five factors could explain the 73.234% of the variance, providing solid evidence for the validity of the questionnaire (Tang & Wen, 2020).

The AMOS 26.0 was applied to conduct the standardised loading of each variable, and the results were examined and illustrated in Table 1. The value of each variable ranged from 0.769 to 0.975, and all the results reached a significant level (when *p* < 0.01). The Average Variance Extracted (AVE) of each dimension (measured altogether by similar variables) ranged from 0.549 to 0.693 (more than the suggested value of 0.5). The Construct Reliability (CR) of each dimension ranged from 0.717 to 0.899 (more than the suggested value of 0.7). The above results indicated the reasonable convergent validity to measure dimensions with existing variables. Furthermore, by comparing the square root of each variable’s AVE value and the correlation coefficient among variables, the high discriminant validity was confirmed.

To further analysis, Amos 26.0 was applied to conduct the confirmatory factor analysis (CFA), and the overall model fitness was tested. The model fit showed that X^2^ = 414.68, d*f* = 163, X^2^/d*f* = 2.544, GFI = 0.803, NFI = 0.859, CFI = 0.832, and RMSEA = 0.032. Those results demonstrated the model was generally well-fitted and deemed suitable for subsequent analysis.

### 4.2. Correlation Analysis

The results of data description and correlation analysis are illustrated in Table 2. The correlation analysis showed that the correlation coefficient between each pair of variables ranged from −0.278 to 0.438, with all relationships being highly significant at the level of *p* < 0.01. As most of the correlation coefficients were significant, it might have a potential multi-collinearity problem. The Variance Inflation Factor (VIF) was tested as each dimension ranged from 1.916 to 3.644 (less than the suggested value of 10), indicating that there is no problem of multi-collinearity among all dimensions. Additionally, the correlation coefficient between willingness to participate and perceived cost was not significant at any level, so Hypothesis H1b was not supported. Other hypotheses were tentatively confirmed.

### 4.3. Structural Equation Model Assessment

The structural equation model (SEM) was often used to consider the complicated relationships among different variables. This research applied maximum likelihood estimation to test the hypotheses, as SEM has been indicated to be efficient by the aforementioned analysis. The results demonstrated that X^2^ = 507.419, d*f* = 163, X^2^/d*f* = 3.113, GFI = 0.837, NFI = 0.859, CFI = 0.869, and RMSEA = 0.035, indicating an appropriate fit that meets the predetermined criteria. As illustrated in Table 3, H1a, H2a, H2b, and H3 were significantly supported.

### 4.4. Analysis of Mediating and Moderating Effects

The PROCESS procedure of SPSS was developed by Hayes to examine the potential moderating and mediating effects existing in the construct model (Hayes, 2012). In this research, the PROCESS was employed to carry out the conditional process model analysis, and to address the potential neutralisation of mediating effects when moderating variables might exhibit effects at high and low levels. As per the previous expectation, the mediating model with moderation aligns with the research hypotheses (Hayes, 2012).

In the analysis of the mediating effect, it is in the co-existence of the moderating effect. As posited by Hypotheses 3, Model 4 with the bootstrap inference of PROCESS was employed for the estimation (Wicker et al., 2012; Wicker & Hallmann, 2013). Considering the sample size was 572 respondents, which met the recommended minimum sample size, a bootstrap size of 5000 was used (Hayes, 2012). The objective of mediating effect analysis is to calculate the direct and indirect effect of each variable (Hayes, 2012), and in this case, the embodied experience. As shown in Table 4, the index of LLCI and ULCI indicated the minimum and maximum values of confidence intervals. On the left side, Hypothesis 4a is illustrated, showing the mediating effect of embodied experience between perceived value and willingness to participate. On the right side, Hypothesis 4b is illustrated, showing the mediating effect of embodied experience between perceived cost and willingness to participate. All the LLCI and ULCI were above zero for each relation, and at the significance level of *p* < 0.001. The embodied experience accounted for 58.2% of the relationship between perceived value and willingness to participate, while it also accounted for 58.2% of the relationship between perceived cost and willingness to participate. These data provided support for Hypotheses 4a and 4b.

**Figure 2:**
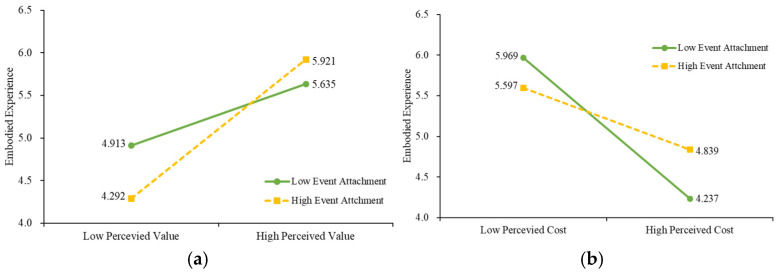
Moderated effect of event attachment. (**a**) Moderating role of event attachment in the relationship between perceived value and embodied experience. (**b**) Moderating role of event attachment in the relationship between perceived cost and embodied experience.

For the moderating effect analysis, considering the relation in the theoretical framework, Model 7 with the bootstrap inference of PROCESS was applied for the moderated mediation model for Hypotheses 5a and 5b (Hayes, 2012). Model 7 was used to further analyse the complex effects when the mediating and moderating effects coexist at the same time (Hayes, 2012). Model 7 focused on the moderating effect when the moderator influenced the relation between independent variables and the mediating variables (Hayes, 2012). Considering the sample size was 572 respondents, which met the recommended minimum sample size, a bootstrap size of 5000 was used (Hayes, 2012; Tang & Wen, 2020). As shown in Table 5, the results indicated that event attachment exhibited a significant moderating effect on the association among perceived value, perceived cost, embodied experience, and willingness to participate. Different levels of event attachment performed differently, as shown in Figure 2, which presents simple slopes to further explore the nature of the moderating interaction.

Table 5 demonstrates the moderating impact of event attachment. The results of the hierarchy multiple regression model indicated that event attachment had a significant impact on embodied experience when the independent variable was perceived value (t = 8. 879, *p* < 0.001) and when the independent variable was perceived cost (t = 6.24, *p* < 0.001). For the internal effects for two lines in the model among perceived value, embodied experience, and willingness to participate, the pivotal role of event attachment was determined. In the comparison of Model 1 (embodied experience as a dependent variable) and Model 2 (willingness to participate as a dependent variable) for the different dependent variable tests, the pronounced alterations in the F value and interaction presence indicated that the strength of event attachment varied considerably at different levels of embodied experience, thereby influencing willingness to participate. The findings supported Hypotheses H5a and H5b.

The simple slope analysis was then tested to see the extent of event attachment on the dependent variables. Figure 2a shows that, when the event attachment was low, the influence between perceived value and embodied experience was lower than when the event attachment was high. Quite the opposite, Figure 2b illustrates the different influence path between perceived cost and embodied experience. It can be seen that when the event attachment increased, it would decrease the negative feelings towards the embodied experience.

## 5. Discussion

Firstly, this research revealed the significant correlation between perceived value, perceived cost, and willingness to participate. While the findings suggested that the perceived value had a strongly positive influence on tourists’ willingness to participate, the perceived cost had a strongly negative influence on their willingness to participate. This is in accordance with the conclusion of previous research which indicated that the perception of the tourism activity could have a significant effect on their experience (Farkic, 2022; Y. Ouyang et al., 2021). Notably, a similar pattern in studies of spectator sports events showed the robustness of this relationship, and the findings strengthened the relationship among different sporting contexts (Wicker et al., 2012). However, the perceived value and perceived cost might vary in the different stages of decision-making. Interviews of some runners indicated that perceived value often exerts its influence on willingness to participate long before the event, while perceived cost frequently affects the willingness to participate in the closer proximity to the event. For instance, the commitment to health maintenance, as exemplified by a runner’s pursuit of long-term running habits, is a behaviour that is not easily altered. However, should the cost of participation be excessively high, the individual may be compelled to withdraw from a certain event, rather than relinquishing their running habit and participating in other events altogether (J. Zhang & Dai, 2023). When the tourists find that they could gain more benefits, for example, maintaining health, socialising, enjoying the city, etc., they would be more willing to join the event, and generate a more integrated experience. Conversely, should the runners perceive the training to be a waste of time or the financial outlay to be excessive, they may be disinclined to participate in the event (Wicker et al., 2012). Even if they do decide to take part, they are unlikely to gain a significant embodied experience, as they will not be able to fully engage with the activity to the same extent as others. This finding is consistent with previous research, illustrating that the negative attitude necessarily hinders tourists’ willingness to participate in the activities (Ahn & Kwon, 2020; Wang et al., 2023; Wicker et al., 2012).

Secondly, this research has paid attention to stressing the importance of the embodied experience as an important part of the event involvement process. In consideration of the assumption that the behaviour exhibited at the event is presumed to be more experiential and dynamic, given the involvement of the body and mind, in comparison to previous research, this finding extends the application scale and focuses more on the individual experience and perception, and leads to further exploration of personal being (Farkic, 2022; Y. Ouyang et al., 2021). According to the previous research, this finding matches the trend that tourists no longer merely seek natural views, but rather crave immersive and memorable experiences (Shapiro et al., 2019). When runners in the Guangzhou Marathon are fully immersed in the race, they experience a seamless integration of the body and mind, losing track of time and external distractions. This phenomenon is consistent with the flow theory of positive psychology as their physical exertion, the rhythm of their breathing, and the feel of the pavement beneath their feet all contribute to a unique embodied experience (Xiang & Cheah, 2023; Jackman et al., 2021). More importantly, the social interactions during events among different bodies and the environment enhance the overall experience and satisfaction of participants (Getz & Page, 2019; Andrades & Dimanche, 2018). The relationship between the embodied experience and willingness to participate was also discussed in this research, and it turned out that the more tourists immersed themselves to have an embodied experience, the more they would be willing to participate in the event (Coghlan & Filo, 2013; Y. Ouyang et al., 2021). This is aligned with the previous study that tourists who had a positive embodied experience during a sports event were more likely to recommend the event to others, return for future editions, and engage in related activities (Coghlan & Filo, 2013; Wicker & Hallmann, 2013).

Thirdly, this research highlighted the significant function of event attachment when it is connected with other contributing factors in the MPSE context. In the realm of event studies, event attachment has emerged as a crucial construct that intertwines with multiple elements to shape the overall event experience (Alimamy & Al-Imamy, 2022; J. Zhang & Dai, 2023). In the findings, event attachment was identified as a moderator in the process of tourist behaviour formation, exerting a direct influence on the relationship between the embodied experience and perceived value and willingness to participate. It has been documented that tourists who had a high level of event attachment were more inclined to immerse themselves to obtain the bodily and mental experience and form unforgettable event memories (Wood & Kinnunen, 2020). The existing literature on tourist event attachment suggested that the attachment was always associated with emotional engagement and a sense of connection with the event (Tsaur et al., 2019). The findings showed that when considering its connection with perceived value, the event attachment could amplify the perceived value that tourists derive from an MPSE (Ferreira et al., 2023). For example, in the context of the Guangzhou Marathon, runners who have a strong attachment to the event are more likely to notice and appreciate the meticulous organisation of the race, such as the variety and quality of refreshment stations, the scenic beauty of the racecourse, and the enthusiastic support from volunteers. Moreover, the findings also showed that event attachment could decrease the negative side of perceived cost and act in a mitigating role for tourists (Zheng et al., 2022). After a decade of Guangzhou Marathon development, the attachment to the event is strongly associated with the host city. As some of the respondents noted, the attachment to the event was deepening due to the bonding to Guangzhou tourism destinations, the Lingnan culture of the city, and the traditional Canton cuisine. This was related to the previous research that emotion and memory could increase the experience (Wood & Kinnunen, 2020).

Fourthly, as this research is a case study for the deep exploration of the MPSE context, the findings hold similar flaws to any other case study. The Guangzhou Marathon is the research case used to typically represent the MPSEs; however, there are numerous other types of MPSEs. Like other case studies, the findings might encounter the challenge of limited conceptual generalisation (J. Zhang & Dai, 2023). It is also noted that a comparative analysis of the embodied behaviour in different types of event participants and tourists is needed for further investigation. Such comparisons would facilitate a more comprehensive understanding of sensory and emotional engagement in diverse events. Multiple comparative case studies are recommended to enhance the credibility and generalisability of the embodied behaviour findings. Furthermore, it is confirmed that government policy and commercial background often influence the environment for event development. This research took the marathon event in China and found the cultural context factors influencing the organisation of the event. Different from traditional spectator sports events, the development of MPSEs is often shaped by distinctive socio-cultural factors (Risitano et al., 2017). Future research considering the contextual influences in greater detail would be beneficial. The comparison of marathons or MPSEs in different cultures or countries would provide a more nuanced understanding of embodied behaviour in both Eastern and Western views. Also, the survey of runners in this research lasted for two sessions of the Guangzhou Marathon 2023 and 2024, implying a small longitudinal study. Yet, this research did not account for changes in attitudes and experiences over more than two sessions, as the existing literature showed that the runners’ involvement changed over time and in accordance with the running times (Wicker et al., 2012; Wicker & Hallmann, 2013). MPSEs were deemed to have a long-lasting impact on participants’ daily routines and behaviours, so longitudinal studies should be conducted to gain a deeper view of the long-term significance for the embodied behaviours in events. Moreover, the evolution of tourists’ behaviours, attitudes, and ultimately their well-being should also provide invaluable insights from an individual perspective and inform the design and management of experiences for MPSE organisers.

## 6. Conclusions and Implication

### 6.1. Conclusions

To conclude, this research used self-report data with greater credibility and provided empirical evidence for the embodied behaviour of MPSE tourists. The findings demonstrated the mechanism of the experience formation process through the path of perception (previous expectation and impact)—bodily and emotional involvement—feedback. This research advanced the knowledge of MPSEs in the Chinese context by bringing out a specific case and providing insight into embodied cognition theory literature.

This research set the growing significance of MPSEs in global tourism, with the Guangzhou Marathon serving as a prime example. Such events have become not only platforms for athletic competition but also major tourist attractions, attracting a diverse range of participants and spectators. The findings made an invaluable contribution to the field of event study by applying the embodied cognition theory to understand the complex interplay of tourists’ behaviour. The study established a framework for analysing the relationship between perceived value, perceived cost, and embodied experience, event attachment, and willingness to participate. This framework facilitates a comprehensive understanding of how embodied behaviour was formed, and its antecedents and consequences factors. The utilisation of a questionnaire, together with interviews and field observation, permitted the exploration of embodied behaviour in the context of tourism activity, using the findings regarding mass participant sports events. The results confirmed and extended previous research on event perception, embodied behaviour, and willingness to participate. Furthermore, the moderating role of event attachment was identified in the relationship between event perception and embodied experience.

Moreover, this research addressed the importance of embodied behaviour, including the body–emotional involvement in tourists’ engagement in MPSEs. As the marathon is a sport with more intensive body movement than any other sport, the concept of the embodied experience is situated at the core of the runner experience in the marathon event. Bodily, sensory, and emotional engagement has a considerable impact on the overall event experience. Even though the experience could be negative in marathon events as the body is facing limits and challenges, the power of the body and emotion could help to form lasting memories and influence the behaviour.

To make tourists’ participation in MPSEs more sustainable in the future, the findings suggested that event organisations focus on strengthening the positive interaction and mitigating the negative interaction among variables. This can be achieved by creating more opportunities for social interaction and community building. The necessity for policymakers to provide support and promotion for such events, with a view to enhancing local tourism economies, is also addressed. Exploring the role of cultural differences in shaping experience in different events must be considered for event organisation.

### 6.2. Managerial Implications

This research yielded actionable and sustainable practices that have the potential to be implemented in the domain of event management. Firstly, it is imperative to acknowledge that, during the initial planning and design stages of public sports events, the sensory and emotional experiences of participants should be regarded as a fundamental objective. The creation of an event environment characterised by multi-sensory engagement, in conjunction with the emotional resonance points provided by event organisers, has the potential to engender elevated levels of satisfaction and foster long-term loyalty among tourists.

Secondly, event management should pay attention to embodied behaviour with experience and attachment. This systematic approach could be used to assess runners’ perceptions and analyse the embodied behaviour. Organisers could identify patterns and trends that shed light on what aspects of the event enhance or detract from the runners’ experience and attachment. In order to increase tourists’ intention to participate in the event, it is recommended that organisers consider the bodily and emotional factors involved in the behavioural process. Designing more interactive emotionally engaging activities to create a bond with the event is critical, and the emotional engagement also levels up the recommending likelihood for tourists.

Thirdly, governments, public sectors, and event originations should prioritise tourists’ experience, and respect their behaviour when carrying out MPSEs and event-related activities. This lays the foundation for the making of a truly remarkable MPSE. It not only guarantees the immediate enjoyment of the participants but also fosters the long-term growth and reputation of the event, attracting more tourists. Working in harmony with different organising sections could also ensure the sustainable development of the local sports and tourism ecosystem.

The implementation of these strategies in future event management enables event organisers to adopt a more inclusive and participant-centric approach to event tourism and sports events, which benefits not only tourists but also other types of event participants.

## Figures and Tables

**Table 1:** Confirmatory factor analysis.

Variable/Item	Standardised Loading	AVE	CR
Perceived Value		0.619	0.765
PV1 Increase physical involvement and health	0.804		
PV2 Gain benefits from the participation	0.769		
Perceived Cost		0.561	0.717
PC1 Spend more time/money preparing for the event	0.699		
PC2 Perception of increased risk during the event	0.795		
Event Attachment		0.549	0.782
EA1 Sense of identification with the event	0.638		
EA2 Importance of participating in the event	0.712		
EA3 Emotions towards the event rather than other events	0.856		
Embodied Experience		0.567	0.887
EE1 More sensory experience (taste/smell, etc.) in the event	0.751		
EE2 More interaction with others in the event	0.655		
EE3 More body and environment interaction in the event	0.824		
EE4 Strong body involvement in the event experience	0.740		
EE5 Strong body–emotion connection in the event	0.733		
EE6 Strong emotion/mind changes in the event experience	0.802		
Willingness to Participate		0.693	0.899
WP1 Willingness to participate before the existing event	0.995		
WP2 Pay more attention to training for the existing event	0.801		
WP3 Readiness of knowledge to the existing event	0.704		
WP4 Willingness to be staff/volunteer of the existing event	0.803		

**Table 2:** Correlation analysis.

Construct/Dimension	Mean	Standard Deviation	1	2	3	4	5	VIF
1. Perceived Value	4.816	1.374	1.000					3.148
2. Perceived Cost	4.437	1.080	0.132 *	1.000				1.916
3. Embodied Experience	5.126	0.982	0.301 **	−0.278 **	1.000			4.197
4. Willingness to Participate	5.352	1.189	0.414 **	0.060	0.316 **	1.000		3.644
5. Event Attachment	4.984	1.086	0.294 **	0.394 **	0.438 **	0.308 **	1.000	3.642

*: *p* < 0.05, Slight significance, two-tailed. **: *p* < 0.01, High significance, two-tailed.

**Table 3:** Hypothesis test results of structural equation model.

Hypothesis	Standard Coefficient	S.E.	C.R.	*p*	Results
**H1a** Perceived Value→Willingness to Participate	0.579	0.040	4.505	***	Supported
**H1b** Perceived Cost→Willingness to Participate	-	-	-	-	Unsupported
**H2a** Perceived Value→Embodied Experience	0.148	0.027	5.538	***	Supported
**H2b** Perceived Cost→Embodied Experience	−0.290	0.034	8.550	***	Supported
**H3** Embodied Experience→Willingness to Participate	0.658	0.099	8.349	***	Supported

***: *p* < 0.001, High significance, two-tailed.

**Table 4:** Hierarchical multiple regression and mediating effect.

Variables	SE		t	*p*	SE		t	*p*
Constant	0.1412		2.5838	**	0.5729		1.9093	***
Perceived Value	0.0206		5.1488	***	-		-	-
Perceived Cost	-		-	-	0.0295		1.5161	***
Embodied Experience	0.0392		2.4801	***	0.0338		3.2673	**
Embodied Experience × Perceived Value	0.0513		2.2129	***	-		-	-
Embodied Experience × Perceived Cost	-		-	-	0.0372		2.4673	***
	**Effect**	**SE**	**LLCI**	**ULCI**	**Effect**	**SE**	**LLCI**	**ULCI**
Total Effect	0.6418	0.0243	0.5940	0.6896	0.6376	0.0376	0.5637	0.7115
Direct Effect	0.2680	0.0280	0.2130	0.3230	0.1105	0.0338	0.0441	0.1769
Mediating Effect	0.3738	0.0319	0.3112	0.4360	0.5271	0.0432	0.4452	0.6120

**: *p* < 0.01, High significance, two-tailed. ***: *p* < 0.001, High significance, two-tailed.

**Table 5:** Hierarchical multiple regression and moderating effect.

Variable	Model 1 (Embodied Experience)				Model 2 (Willingness to Participate)			
	β	SE	t	*p*	β	SE	t	*p*
Constant	5.169	0.026	6.274	***	1.656	0.203	8.171	***
Perceived Value	0.165	0.027	6.238	***	0.268	0.028	9.567	***
Embodied Experience	-	-	-	-	0.721	0.039	8.402	***
Event Attachment	0.541	0.033	6.405	***	-	-	-	-
Perceived Value × Event Attachment	0.056	0.011	5.375	***	-	-	-	-
R^2^	0.830				0.847			
Adjusted R^2^	0.689				0.718			
F	20.205 ***				23.066 ***			
Constant	5.160	0.025	6.681	***	0.656	0.193	3.404	***
Perceived Cost	0.226	0.027	8.454	***	0.110	0.034	3.267	***
Embodied Experience	-	-	-	-	0.916	0.037	4.627	***
Event Attachment	0.573	0.027	2.012	***	-	-	-	-
Perceived Cost × Event Attachment	−0.049	0.014	−3.530	***	-	-	-	-
R^2^	0.829				0.824			
Adjusted R^2^	0.688				0.678			
F	17.408 ***				19.746 ***			

***: *p* < 0.001, High significance, two-tailed.

## Data Availability

Data are available from the corresponding author on reasonable request.
